# The Interplay Between Sleep Disturbance, Glymphatic Dysfunction and Altered Brain Networks in Internet Gaming Disorder: A Multimodal Neuroimaging Study

**DOI:** 10.1111/adb.70175

**Published:** 2026-07-01

**Authors:** Jiawen Tian, Hui Zhang, Xinyu Wang, Hongyu Zhang, Longyao Ma, Bohui Mei, Mengzhe Zhang, Yan Lang, Yarui Wei, Shaoqiang Han, Qingqing Lv, Yong Zhang

**Affiliations:** ^1^ Department of Magnetic Resonance Imaging the First Affiliated Hospital of Zhengzhou University Henan China; ^2^ Zhengzhou Key Laboratory of Brain Function and Cognitive Magnetic Resonance Imaging Henan China; ^3^ Henan Engineering Technology Research Center for Detection and Application of Brain Function Henan China; ^4^ Henan Engineering Research Center of Medical Imaging Intelligent Diagnosis and Treatment Henan China; ^5^ Henan Key Laboratory of Imaging Intelligence Research Henan China; ^6^ Henan Engineering Research Center of Brain Function Development and Application Henan China; ^7^ Department of Psychiatry the First Affiliated Hospital of Zhengzhou University Henan China; ^8^ Department of Radiology the Third Affiliated Hospital of Zhengzhou University Henan China

**Keywords:** DTI‐ALPS, functional connectivity, Internet gaming disorder, middle frontal gyrus, sleep disturbances

## Abstract

Sleep disturbances are common in individuals with Internet gaming disorder (IGD), yet the neurobiological links between poor sleep quality and addictive symptoms remain unclear. The glymphatic system contributes to cerebrospinal fluid–interstitial fluid exchange and metabolic waste clearance, processes that are closely related to sleep physiology. Diffusion tensor image analysis along the perivascular space (DTI‐ALPS) provides a non‐invasive MRI‐based marker related to glymphatic system function. In parallel, middle frontal gyrus (MFG)–centred functional connectivity may reflect prefrontal network alterations associated with cognitive control and addiction severity in IGD. The study included 30 individuals with IGD and 37 demographically matched healthy controls (HCs). All participants underwent diffusion tensor imaging and resting‐state functional MRI. DTI‐ALPS was used to assess glymphatic system–related function. Resting‐state functional connectivity analysis was performed using the MFG as the seed region. Group differences in ALPS indices were tested while controlling for age, education years and mean framewise displacement. Within the IGD group, correlation analyses were conducted to examine associations among ALPS indices, PSQI scores, MFG‐centred functional connectivity and IAT scores. Individuals with IGD showed significantly lower mean and left ALPS indices than HCs. The right ALPS index showed a non‐significant trend towards reduction. Within the IGD group, higher PSQI scores were associated with lower mean, left and right ALPS indices, suggesting that poorer subjective sleep quality was related to reduced glymphatic system–related function. MFG‐centred functional connectivity analysis showed increased connectivity mainly involving medial and superior frontal regions and decreased connectivity involving subcortical regions. Stronger MFG‐centred functional connectivity was positively correlated with IAT scores, indicating an association with greater addiction severity. This study integrates DTI‐ALPS and MFG‐centred resting‐state functional connectivity to examine sleep‐related glymphatic alterations and prefrontal network changes in IGD. Lower ALPS indices were associated with poorer sleep quality, whereas stronger MFG‐centred connectivity was associated with greater addiction severity. These findings support a potential sleep–glymphatic–prefrontal network framework for understanding IGD. Longitudinal and interventional studies are needed to clarify the directionality of these associations.

## Graphical abstract

This multimodal neuroimaging study explores the neurobiological mechanisms of Internet Gaming Disorder (IGD) by integrating DTI–ALPS and resting–state functional connectivity. The findings reveal that individuals with IGD exhibit reduced glymphatic system–related function, which is significantly associated with poorer subjective sleep quality. Furthermore, altered middle frontal gyrus (MFG–centred functional connectivity is positively correlated with addiction severity. Together, these results support a novel sleep–glymphatic–prefrontal network framework for understanding IGD.

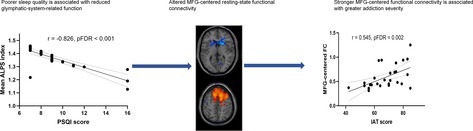

## 1 Introduction

With the widespread penetration of the internet, Internet gaming disorder (IGD) has emerged as a global public health issue [1]. Clinical observations and previous studies have shown that individuals with IGD commonly experience sleep disturbances, including interrupted sleep, reduced sleep duration and diminished sleep quality [2]. These sleep problems may exacerbate emotional distress and cognitive difficulties, thereby impairing daily functioning and recovery. However, the neural mechanisms underlying the comorbidity between IGD and sleep disturbances remain poorly understood.

The glymphatic system facilitates cerebrospinal fluid–interstitial fluid exchange and contributes to the clearance of metabolic waste from the brain [3]. Sleep has been shown to enhance metabolite clearance through this pathway, suggesting that sleep plays an important role in maintaining brain metabolic homoeostasis [4]. Conversely, sleep disruption may compromise glymphatic clearance and contribute to neural dysfunction. Clinical evidence also suggests that glymphatic dysfunction is involved in various neurological disorders and is associated with poor sleep quality [5]. Diffusion tensor image analysis along the perivascular space (DTI‐ALPS) provides a non‐invasive MRI‐based index for assessing glymphatic system function [6, 7]. Previous studies have further linked DTI‐ALPS indices with sleep quality, cognitive performance and brain structural alterations [8]. Therefore, DTI‐ALPS may provide a useful imaging marker for investigating whether sleep disturbances in IGD are associated with impaired glymphatic function.

Resting‐state functional magnetic resonance imaging (rs‐fMRI) studies have shown that individuals with IGD exhibit abnormal functional connectivity in brain networks involved in cognitive control, reward processing and self‐referential processing [9, 10, 11, 12]. Among prefrontal regions, the middle frontal gyrus (MFG) is particularly relevant to IGD because it is a key region involved in executive control, response inhibition, decision‐making and top‐down regulation of reward‐driven behaviour. Previous neuroimaging studies have implicated prefrontal network dysfunction and altered MFG‐related connectivity in IGD‐related impulsivity, impaired cognitive control and addictive gaming behaviour [9, 10, 11, 12]. Therefore, the MFG was selected as the seed region in the present study to examine whether prefrontal network alterations are associated with addiction severity and sleep‐related glymphatic dysfunction in IGD.

However, direct imaging evidence remains limited regarding whether sleep disturbances in IGD are associated with glymphatic system dysfunction and altered functional connectivity in core brain regions. Moreover, previous studies have rarely integrated functional brain network analysis with imaging markers of the brain's waste clearance system. This gap limits our understanding of how sleep‐related physiological dysfunction and brain network abnormalities may jointly contribute to the clinical manifestations of IGD.

Based on this background, we hypothesized that sleep disturbances in IGD may be associated with reduced glymphatic system function, reflected by lower DTI‐ALPS indices and altered MFG‐centred functional connectivity in prefrontal networks involved in cognitive control. Furthermore, these neuroimaging alterations may be associated with clinical severity, including sleep quality and addiction severity. Therefore, the present study integrated DTI‐ALPS and MFG‐centred resting‐state functional connectivity analyses to investigate the relationship among sleep disturbances, glymphatic dysfunction and altered brain networks in individuals with IGD. This multimodal approach may provide a more comprehensive perspective on the ‘sleep–glymphatic–brain network’ pathway underlying IGD.

## 2 Materials and Methods

### 2.1 Participants

A total of 67 participants, including 30 individuals with IGD and 37 demographically matched HCs, were recruited for this study. IGD was diagnosed by two psychiatrists utilizing the fifth edition of the Diagnostic and Statistical Manual of Mental Disorders (DSM‐5) [13]. The severity of IGD was assessed with the Young's Internet Addiction Test (IAT) [14]. All participants were right‐handed.

The inclusion criteria for IGDs were as follows: (1) compliance with DSM‐5 diagnostic criteria for IGD; (2) IAT scores ≧ 50 points; (3) no previous anti‐neuropathy medication; (4) not taken any sedative, sleeping, analgesic or narcotic drugs; (5) no previous severe craniocerebral trauma or organic brain disease; and (6) no substance abuse or dependence history other than IGDs (nicotine, alcohol, etc.). The inclusion criteria for HCs were as follows: (1) physical and mental health, without any mental illness or neurological disease; (2) IAT scores < 40 points; and (3) no prior history of substance abuse or dependence. Exclusion criteria were as follows: (1) mentally retarded individuals; (2) contraindications to MRI scanning such as metal braces, pacemakers and claustrophobia; (3) pregnant or nursing women; and (4) psychiatric disorders or hereditary familial disorders.

For individuals with IGD, clinical characteristics were further assessed using the Pittsburgh Sleep Quality Index (PSQI) [15], Hamilton Anxiety Scale (HAMA) [16] and Hamilton Depression Scale (HAMD) [17]. These scales were used to characterize anxiety symptoms, depressive symptoms and subjective sleep quality in the IGD group. For HCs, IAT was used only as a screening tool to exclude potential IGD, and HAMA, HAMD and PSQI were not systematically administered. Therefore, HC clinical scale scores were not included in Table 1 or between‐group comparisons.

**TABLE 1:** Demographic and clinical characteristics of individuals with IGD and healthy controls.

Variable	IGD (*n* = 30)	HC (*n* = 37)	*p*
Age, years	15.00 ± 2.21	15.95 ± 5.98	0.378
Education, years	9.23 ± 1.91	9.22 ± 4.18	0.982
Mean FD, mm	0.14 ± 0.04	0.13 ± 0.04	0.519
IAT score	65.03 ± 10.77	—	—
HAMD score	21.90 ± 12.00	—	—
HAMA score	14.37 ± 11.00	—	—
PSQI score	9.20 ± 2.70	—	—

*Note:* Data are presented as mean ± standard deviation. Age, education years and mean FD were compared between the IGD and HC groups. IAT was used as a screening tool for HCs but was not retained as a clinical variable for subsequent statistical analyses. HAMA, HAMD and PSQI were administered only to individuals with IGD for clinical characterization and brain–behaviour association analyses; therefore, corresponding HC values and between‐group comparisons are not available.

Abbreviations: FD, framewise displacement; HAMA, Hamilton Anxiety Scale; HAMD, Hamilton Depression Scale; HC, healthy controls; IAT, Young's Internet Addiction Test; IGD, Internet gaming disorder; PSQI, Pittsburgh Sleep Quality Index.

All subjects were informed of the purpose, procedure, contraindications and possible discomfort of the study before the examination, and the informed consent was signed by themselves or their guardians and approved by the Ethics Committee of the First Affiliated Hospital of Zhengzhou University. The study was conducted in accordance with the Declaration of Helsinki.

### 2.2 MRI Data Acquisition

Magnetic resonance data for all subjects were acquired using the Siemens Prisma 3.0‐T magnetic resonance scanner with a 64‐channel receiver array head coil. The soft cushion was utilized on each side of the neck to keep the subject's head braked, and earplugs were applied to mitigate scanning noise.

Standard MRI sequences include axial T1‐weighted imaging (T1WI), axial T2‐weighted imaging (T2WI), axial T2‐weighted FLAIR (T2‐FLAIR) and axial diffusion–weighted imaging (DWI) (b = 0, 1000 s/mm^2^). These standard sequences are primarily used to examine whether subjects have any undetected intracranial organic lesions. Subjects were scanned using conventional sequences with the following parameters: echo time (TE) = 93 ms, repetition time (TR) = 3800 ms, slice thickness = 5 mm, number of slices = 20, inter‐slice spacing = 1 mm, matrix = 256 × 256.

High‐resolution T1‐weighted structural images were acquired using a 3D magnetization‐prepared rapid gradient echo sequence with the following parameters: TR = 2300 ms; TE = 2.32 ms; flip angle = 9°; matrix size = 256 × 256; slice thickness = 0.9 mm; slice gap = 0 mm; number of slices = 176; voxel size = 0.9 × 0.9 × 0.9 mm^3^; and field of view (FOV) = 240 × 240 mm^2^.

DTI sequence scan parameters are as follows: TR = 9200 ms; TE = 72 ms; inversion time (TI) = 900 ms; b values = 1000 s/mm^2^; number of diffusion gradient directions = 64; slice thickness = 2.0 mm; slice spacing = 0 mm; number of slices = 70; flip angle = 9°; FOV = 256 × 256 mm; matrix size = 128 × 128.

Functional MRI data were acquired using an echo‐planar imaging pulse sequence with the following parameters: TR/TE = 1000 ms/30 ms, flip angle = 70°, matrix size = 64 × 64, slice thickness = 2.2 mm, slice gap = 0.5 mm, number of slices = 52, voxel size = 2 × 2 × 2 mm^3^ and FOV = 220 × 220 mm^2^.

### 2.3 rs‐fMRI Preprocessing and MFG‐Centred Functional Connectivity Analysis

rs‐fMRI data for all subjects were preprocessed using the advanced edition of the Data Processing Assistant for Resting‐State fMRI (DPARSF, v5.0; https://rfmri.org/DPABI) toolkit in MATLAB [18]. The main preprocessing steps were as follows: (1) conversion of data format from DICOM to NIFTI; (2) removal of the first 10 volumes of each functional time series due to MRI signal instability; (3) slice‐timing correction; (4) realignment, with exclusion of subjects showing maximum head motion > 2 mm or rotation > 2°; (5) normalization to the EPI template and resampling to 3 × 3 × 3 mm^3^; (6) removal of linear trends; (7) band‐pass filtering at 0.01–0.08 Hz; and (8) regression of nuisance covariates, including Friston 24 head‐motion parameters [19], white matter signal and cerebrospinal fluid signal. Global signal regression was not performed. Images with framewise displacement (FD) > 0.2 were scrubbed using third‐order spline fitting to minimize the effect of head motion while preserving temporal information in this frequency band [20].

The seed region for functional connectivity analysis was selected based on its established involvement in IGD. A 5‐mm‐radius sphere was placed in the MFG, centred on the peak coordinate reported by Kim's study (MNI: x = 28, y = 44, z = 32) [21]. Before seed‐based functional connectivity calculation, the normalized functional images were spatially smoothed in MATLAB using an 8‐mm full width at half maximum (FWHM) Gaussian kernel. The mean time series from this spherical seed was correlated with every other voxel in the brain to create individual whole‐brain functional connectivity maps [22].

Group differences in MFG‐centred functional connectivity were assessed using two‐sample *t*‐tests in Statistical Parametric Mapping software (SPM12, http://www.fil.ion.ucl.ac.uk/spm/). Statistical significance was set at voxel‐level *p* < 0.001 and cluster‐level *p* < 0.05, FDR corrected. Significant clusters were anatomically labelled using xjView and the automated anatomical labelling (AAL) atlas [23]. To determine the association between altered functional connectivity and clinical behaviours, Pearson correlation analyses were performed between clinical indicators in the IGD group and altered MFG‐centred functional connectivity values.

### 2.4 DTI Preprocessing and ALPS Index Calculation

The DTI‐ALPS index was calculated using the FSL pipeline, following previously described methods [6, 7, 24, 25, 26]. First, diffusion data were converted from DICOM to NIFTI format. Second, Marchenko–Pastur principal component analysis–based denoising and Gibbs‐ringing correction were performed using the MRtrix3 commands ‘dwidenoise’ and ‘mrdegibbs’ [27]. Third, eddy‐current and head‐motion correction were conducted using the FSL ‘eddy’ command [24]. Fourth, brain extraction was carried out using the FSL ‘bet’ command to remove skull and non‐brain tissues. Fifth, bias‐field correction was performed to reduce intensity inhomogeneity induced by magnetic field inhomogeneity [28]. Then, the fractional anisotropy (FA) map and x‐axis (Dxx), y‐axis (Dyy) and z‐axis (Dzz) diffusivity maps were generated using the FSL ‘dtifit’ command [24].

To enable cross‐subject comparison and standardized ROI placement, individual diffusion maps were registered to the MNI152 standard space. Linear (FLIRT) and non‐linear (FNIRT) registration were performed using FSL, with the b0 image first aligned to the individual T1‐weighted structural image, which was then normalized to the MNI space. The resulting warp fields were applied to the diffusivity maps to bring them into standard space. The regions of interest (ROIs) were defined as 5‐mm‐radius spheres centred at the spatial coordinates as follows: left SCR (116 110, 99), left SLF (128 110, 99), right SCR (64 110, 99) and right SLF (51 110, 99) [7, 26]. The diffusivity values (Dxx, Dyy and Dzz) for the bilateral SLF and SCR were extracted for the DTI‐ALPS calculation by using the following formula [6]:

$$\mathrm{DTI}-\text{ALPS}=\frac{\text{mean}\left(\mathrm{Dx}x_{\text{proj}},\mathrm{Dx}x_{\text{assoc}}\right)}{\text{mean}\left(\mathrm{Dy}y_{\text{proj}},\mathrm{Dz}z_{\text{assoc}}\right)}$$

The ALPS index was calculated for each hemisphere independently (left/right ALPS index), and the final DTI‐ALPS value was obtained by averaging both sides (mean DTI‐ALPS).

### 2.5 Statistical Analyses

Two‐sample *t*‐tests in SPSS were used to compare demographic and clinical characteristics between groups, with the significance threshold set at *p* < 0.05.

For functional connectivity analysis, two‐sample *t*‐tests in SPM12 were used to compare group differences in MFG‐centred functional connectivity. Statistical significance was set at voxel‐level *p* < 0.001 and cluster‐level *p* < 0.05, FDR corrected. Pearson correlation analyses were performed to examine the associations between altered functional connectivity and clinical indicators in the IGD group.

Group differences in the ALPS index were assessed using analysis of covariance (ANCOVA), controlling for age, education years and mean FD. FDR correction was applied across the three ALPS comparisons. Additionally, to test the hypothesis linking glymphatic system function to sleep disturbances, Pearson correlation analysis was conducted between PSQI scores and ALPS index scores within the IGD group.

For the primary brain–behaviour correlation analyses, Pearson correlation analyses were performed within the IGD group. The primary correlation analyses included MFG‐centred functional connectivity versus IAT score, mean ALPS index versus PSQI score, left ALPS index versus PSQI score and right ALPS index versus PSQI score. FDR correction was applied across these four primary correlation analyses.

## 3 Results

### 3.1 Demographic and Clinical Data

No significant group differences were observed in age (*p* = 0.378), education years (*p* = 0.982) or mean FD (*p* = 0.519). IAT, HAMD, HAMA and PSQI scores were available only for the IGD group and were used for clinical characterization and brain–behaviour association analyses. Detailed demographic and clinical characteristics are presented in Table 1.

### 3.2 MFG‐Centred Functional Connectivity Results

#### 3.2.1 Intergroup Differences in MFG‐Centred Functional Connectivity

**FIGURE 1:**
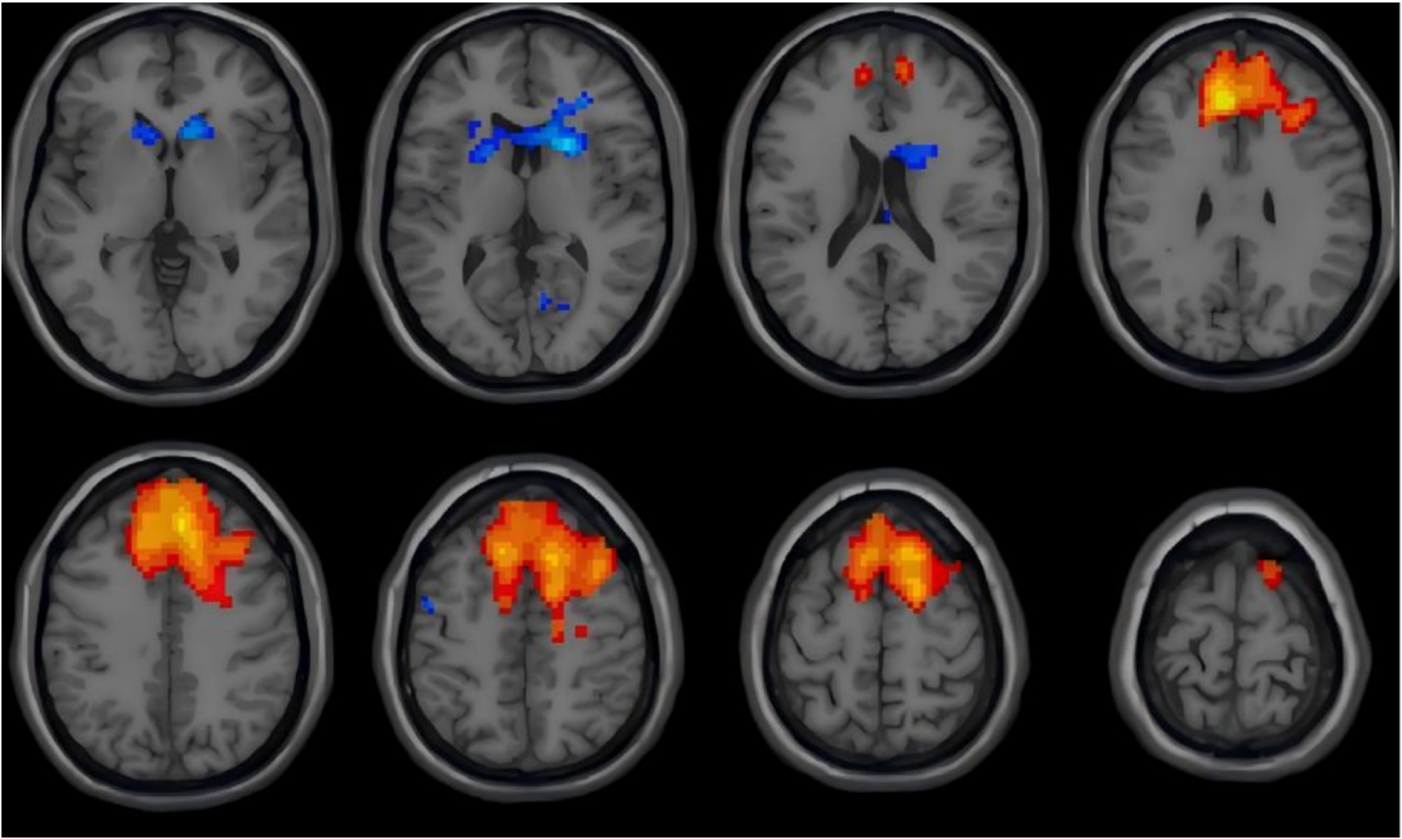
Group differences in MFG‐centred resting‐state functional connectivity between individuals with IGD and healthy controls. Warm‐coloured clusters indicate increased MFG‐centred functional connectivity in individuals with IGD compared with HCs, whereas cool‐coloured clusters indicate decreased MFG‐centred functional connectivity. Results were thresholded at voxel‐level *p* < 0.001 and cluster‐level *p* < 0.05, FDR corrected. FDR, false discovery rate; HC, healthy controls; IGD, Internet gaming disorder; MFG, middle frontal gyrus.

Compared with HCs, individuals with IGD showed a large cluster of increased MFG‐centred functional connectivity, with the cluster‐level peak located in the left superior medial frontal gyrus. AAL‐based anatomical labelling showed that this cluster mainly extended to the bilateral superior frontal gyrus, bilateral superior medial frontal gyrus, right MFG, bilateral supplementary motor area and right middle cingulate gyrus. In contrast, individuals with IGD showed decreased MFG‐centred functional connectivity in a subcortical cluster peaking in the right putamen/lentiform nucleus and mainly involving the bilateral caudate. Results were thresholded at voxel‐level *p* < 0.001 and cluster‐level *p* < 0.05, FDR corrected. Detailed cluster‐level and AAL‐based subregional results are presented in Table 2 and Figure 1.

**TABLE 2:** Cluster‐level and AAL‐based subregional differences in MFG‐centred functional connectivity between individuals with IGD and healthy controls.

Parent cluster	Contrast	Cluster‐level peak label	Cluster‐level peak MNI, x/y/z	Cluster size, voxels	AAL subregion	Hemisphere	Subregion voxels	Regional peak MNI, x/y/z	Regional peak *t*
Cluster 1	IGD > HC	Left superior medial frontal gyrus	−6, 36, 33	3184	Superior frontal gyrus	R	543	17, 16, 47	4.182
Cluster 1	IGD > HC	Left superior medial frontal gyrus	−6, 36, 33	3184	Superior medial frontal gyrus	L	453	−7, 47, 47	3.914
Cluster 1	IGD > HC	Left superior medial frontal gyrus	−6, 36, 33	3184	Middle frontal gyrus	R	419	35, 16, 50	4.152
Cluster 1	IGD > HC	Left superior medial frontal gyrus	−6, 36, 33	3184	Superior medial frontal gyrus	R	372	8, 47, 42	4.405
Cluster 1	IGD > HC	Left superior medial frontal gyrus	−6, 36, 33	3184	Superior frontal gyrus	L	349	−13, 25, 47	4.392
Cluster 1	IGD > HC	Left superior medial frontal gyrus	−6, 36, 33	3184	Supplementary motor area	R	234	13, 16, 62	3.89
Cluster 1	IGD > HC	Left superior medial frontal gyrus	−6, 36, 33	3184	Supplementary motor area	L	206	−1, 22, 64	3.881
Cluster 1	IGD > HC	Left superior medial frontal gyrus	−6, 36, 33	3184	Middle cingulate gyrus	R	109	8, 22, 41	3.969
Cluster 2	IGD < HC	Right putamen/lentiform nucleus	21, 15, 6	485	Caudate	R	125	13, 12, 16	−3.377
Cluster 2	IGD < HC	Right putamen/lentiform nucleus	21, 15, 6	485	Caudate	L	59	−7, 16, 13	−3.495

*Note:* Results were thresholded at voxel‐level *p* < 0.001 and cluster‐level *p* < 0.05, FDR corrected. Cluster‐level peak labels were based on xjView peak‐coordinate labelling; AAL subregions represent the main anatomical subdivisions within each parent cluster. Positive and negative *t* values indicate IGD > HC and IGD < HC, respectively.

Abbreviations: AAL, automated anatomical labelling; FDR, false discovery rate; MFG, middle frontal gyrus; MNI, Montreal Neurological Institute.

### 3.3 ALPS Results

#### 3.3.1 Group Differences in ALPS Indices

**FIGURE 2:**
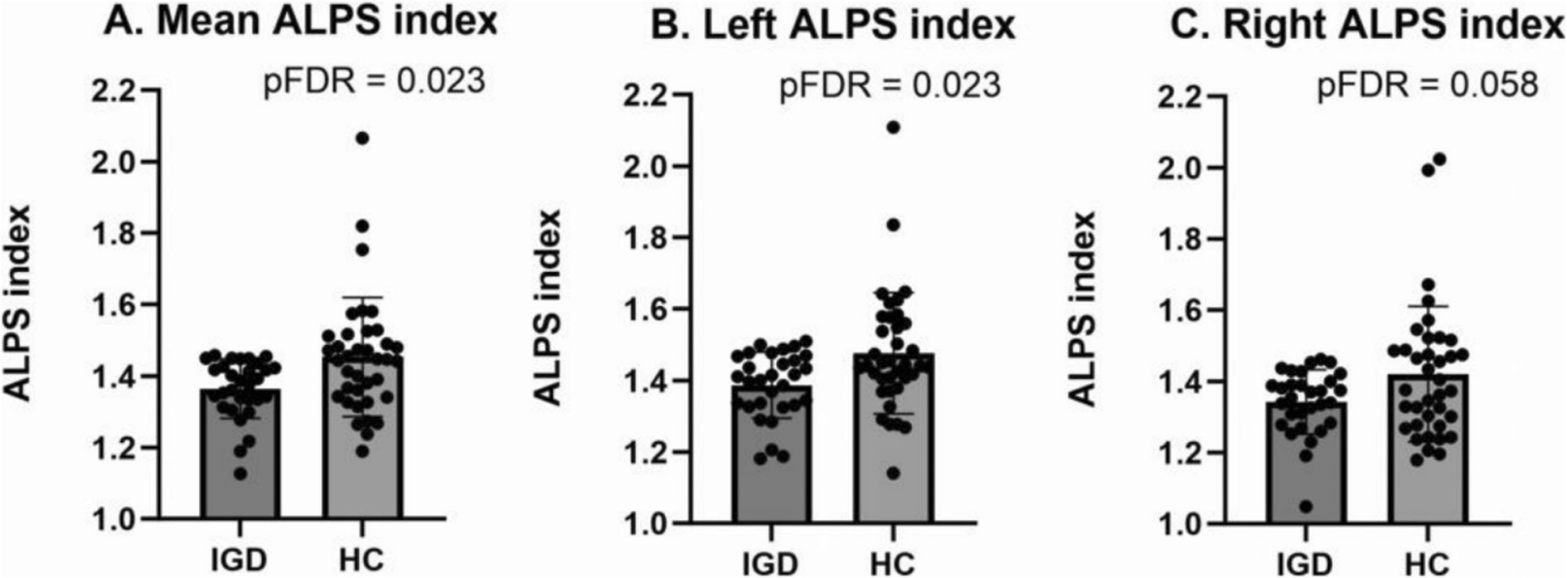
Group differences in ALPS indices between individuals with IGD and healthy controls. Individuals with IGD showed significantly lower mean and left ALPS indices compared with HCs after controlling for age, education years and mean FD, whereas the right ALPS index showed a non‐significant trend. Data are presented as mean ± SD with individual data points. The *p* values represent FDR‐corrected ANCOVA results. ALPS, analysis along the perivascular space; FD, framewise displacement; HC, healthy controls; IGD, Internet gaming disorder.

After controlling for age, education years and mean FD, individuals with IGD showed significantly lower mean ALPS index than HCs (*F*(1,62) = 6.598, *p* = 0.013, FDR‐corrected p = 0.023, partial η^2^ = 0.096). A similar reduction was observed for the left ALPS index (*F*(1,62) = 6.252, *p* = 0.015, FDR‐corrected *p* = 0.023, partial η^2^ = 0.092). The right ALPS index showed a trend towards reduction in the IGD group, but this effect did not reach statistical significance after FDR correction (*F*(1,62) = 3.724, *p* = 0.058, FDR‐corrected *p* = 0.058, partial η^2^ = 0.057). Detailed results are shown in Table 3 and Figure 2.

**TABLE 3:** Group differences in ALPS indices between individuals with IGD and healthy controls.

ALPS measure	IGD (*n* = 30)	HC (*n* = 37)	*F*	*p*	FDR‐corrected *p*	Partial η^2^
Mean ALPS index	1.36 ± 0.08	1.45 ± 0.17	6.598	0.013	0.023	0.096
Left ALPS index	1.39 ± 0.09	1.48 ± 0.17	6.252	0.015	0.023	0.092
Right ALPS index	1.34 ± 0.09	1.42 ± 0.19	3.724	0.058	0.058	0.057

*Note:* Data are presented as mean ± standard deviation. Group differences were assessed using ANCOVA controlling for age, education years and mean framewise displacement. FDR correction was applied across the three ALPS comparisons.

Abbreviations: ALPS, analysis along the perivascular space; FD, framewise displacement; HC, healthy controls; IGD, Internet gaming disorder.

### 3.4 Associations Between ALPS Indices and PSQI Scores

**FIGURE 3:**
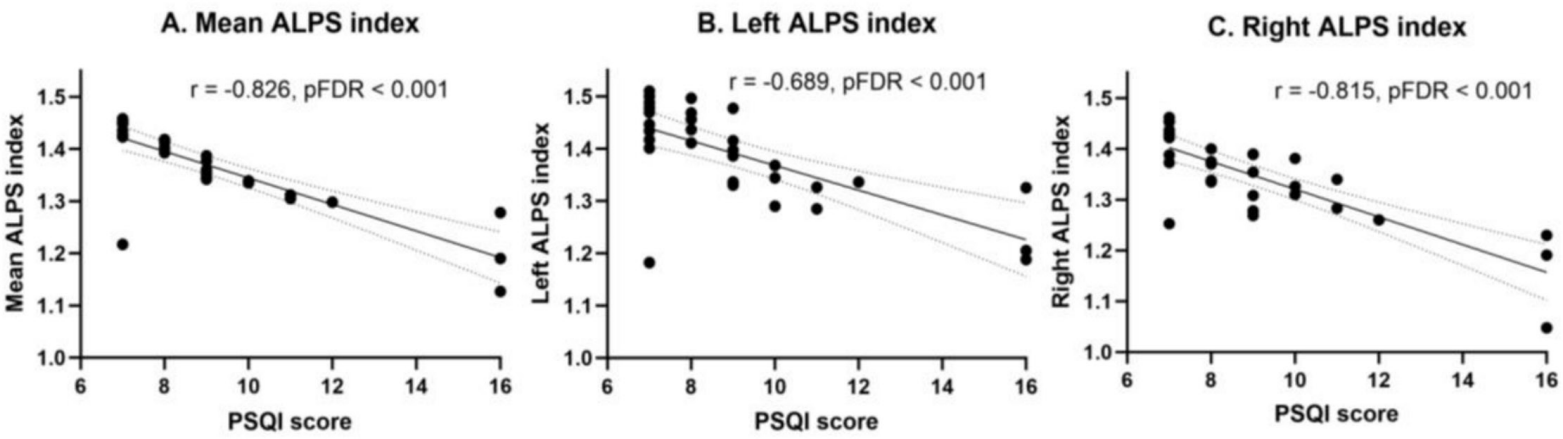
Associations between ALPS indices and PSQI scores in individuals with IGD. Mean, left and right ALPS indices were negatively correlated with PSQI scores in the IGD group. Pearson correlation analyses were performed within the IGD group, and *p* values were corrected using FDR correction across the primary correlation analyses. ALPS, analysis along the perivascular space; FDR, false discovery rate; IGD, Internet gaming disorder; PSQI, Pittsburgh Sleep Quality Index.

Pearson correlation analyses within the IGD group showed that PSQI scores were negatively correlated with the mean ALPS index (*r* = −0.826, uncorrected *p* < 0.001, FDR‐corrected *p* < 0.001), left ALPS index (*r* = −0.689, uncorrected *p* < 0.001, FDR‐corrected *p* < 0.001) and right ALPS index (*r* = −0.815, uncorrected *p* < 0.001, FDR‐corrected *p* < 0.001). These findings suggest that poorer sleep quality was associated with lower ALPS indices in individuals with IGD. Detailed correlation results are summarized in Table 4 and Figure 3.

**TABLE 4:** Correlation analyses between neuroimaging measures and clinical scores in the IGD group.

Analysis	*n*	*r*	Uncorrected *p*	FDR‐corrected *p*
MFG‐centred FC vs. IAT score	30	0.545	0.002	0.002
Mean ALPS index vs. PSQI score	30	−0.826	< 0.001	< 0.001
Left ALPS index vs. PSQI score	30	−0.689	< 0.001	< 0.001
Right ALPS index vs. PSQI score	30	−0.815	< 0.001	< 0.001

*Note:* Pearson correlation analyses were performed within the IGD group. FDR correction was applied across the four primary correlation analyses.

Abbreviations: ALPS, analysis along the perivascular space; FC, functional connectivity; IAT, Young's Internet Addiction Test; IGD, Internet gaming disorder; MFG, middle frontal gyrus; PSQI, Pittsburgh Sleep Quality Index.

### 3.5 Correlation Between MFG‐Centred Functional Connectivity and Clinical Measures

**FIGURE 4:**
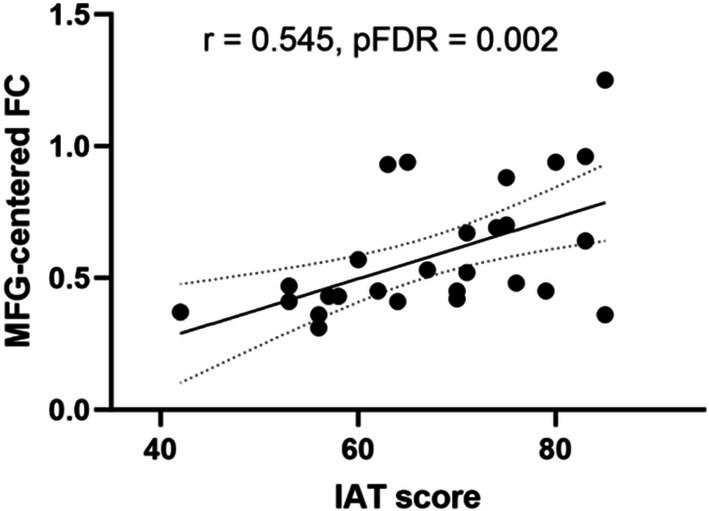
Association between MFG‐centred functional connectivity and IAT score in individuals with IGD. MFG‐centred functional connectivity was positively correlated with IAT scores within the IGD group. Pearson correlation analysis was performed within the IGD group, and the *p* value was corrected using FDR correction across the primary correlation analyses. FC, functional connectivity; FDR, false discovery rate; IAT, Young's Internet Addiction Test; IGD, Internet gaming disorder; MFG, middle frontal gyrus.

Within the IGD group, MFG‐centred functional connectivity was positively correlated with IAT scores (*r* = 0.545, uncorrected *p* = 0.002, FDR‐corrected *p* = 0.002), indicating that stronger MFG‐centred connectivity was associated with greater addiction severity. In exploratory analyses, no significant correlations were observed between MFG‐centred functional connectivity and age, education years, HAMD scores, HAMA scores or PSQI scores (all *p* > 0.05). This association is summarized in Table 4 and shown in Figure 4.

## 4 Discussion

The present study combined DTI‐ALPS and MFG‐centred functional connectivity analyses to examine sleep quality, glymphatic system–related function and brain network alterations in individuals with IGD. Several findings emerged. Individuals with IGD showed lower mean and left ALPS indices than healthy controls, suggesting altered water diffusivity along the perivascular space. Within the IGD group, lower ALPS indices were associated with higher PSQI scores, indicating a relationship between poorer subjective sleep quality and reduced glymphatic system–related function. In parallel, MFG‐centred functional connectivity was altered in IGD. Increased connectivity was mainly observed in medial and superior frontal regions, whereas decreased connectivity involved subcortical regions. Moreover, stronger MFG‐centred functional connectivity was positively correlated with IAT scores. These findings suggest that sleep‐related glymphatic alterations and prefrontal network changes may represent two relevant neuroimaging features of IGD.

The ALPS findings are consistent with the role of the glymphatic system in cerebrospinal fluid–interstitial fluid exchange and metabolic waste clearance [3]. Sleep has been shown to facilitate this clearance pathway, supporting its importance for brain metabolic homoeostasis [4]. In this context, the negative correlations between PSQI scores and ALPS indices suggest that poorer sleep quality in IGD may be linked to reduced glymphatic system–related diffusivity. This interpretation is also in line with clinical evidence connecting glymphatic dysfunction with sleep quality and neurological conditions [5, 8]. However, the present results should be interpreted cautiously. DTI‐ALPS is an indirect imaging marker, and the cross‐sectional design does not allow causal conclusions. Therefore, our findings indicate an association between sleep quality and ALPS indices rather than direct evidence that sleep disturbance causes glymphatic dysfunction.

The present study also extends the application of DTI‐ALPS to behavioural addiction. DTI‐ALPS was originally proposed as a non‐invasive MRI‐based approach for estimating glymphatic system activity [6]. Subsequent studies have supported its reproducibility and reliability across acquisition conditions and scanners [7, 25]. Reduced ALPS indices have been reported in several neurological and psychiatric conditions and have been linked to cognition, amyloid deposition and brain reserve measures [29, 30]. Recent work has also begun to apply DTI‐ALPS to substance‐related disorders, including alcohol use disorder and heroin dependence [31, 32]. Our findings suggest that glymphatic system–related alterations may also be relevant to IGD, particularly in individuals with prominent sleep problems.

MFG‐centred functional connectivity provides another perspective on IGD‐related brain alterations. The MFG is involved in executive control, response inhibition, decision‐making and top‐down regulation of reward‐driven behaviour. These functions are closely related to core clinical features of IGD, including impaired control over gaming and persistent engagement despite negative consequences. Previous rs‐fMRI studies have shown abnormal functional connectivity in cognitive control, reward and default mode–related networks in IGD [9, 10, 11, 12]. In the present study, increased MFG‐centred connectivity with medial and superior frontal regions may reflect altered recruitment of prefrontal control systems during the resting state. This increase may represent a compensatory response, but it may also indicate inefficient or dysregulated prefrontal network functioning. Both possibilities are compatible with previous evidence of impaired executive control and altered control–reward circuit interactions in IGD [10, 33].

The positive association between MFG‐centred functional connectivity and IAT scores further supports the clinical relevance of this prefrontal alteration. Higher IAT scores indicate greater addiction severity. The observed correlation suggests that stronger MFG‐centred connectivity may be related to more severe IGD symptoms. This finding does not establish directionality. Nevertheless, it is consistent with the idea that abnormal prefrontal network communication may be involved in weakened top‐down control over reward‐driven gaming behaviour [10, 21, 33]. In this sense, MFG‐centred connectivity may provide a functional imaging correlate of addiction severity.

The combined use of DTI‐ALPS and functional connectivity is a major feature of this study. These two measures capture different aspects of brain physiology. DTI‐ALPS reflects diffusion properties along the perivascular space and is considered a marker related to glymphatic function. Functional connectivity reflects intrinsic communication between brain regions. In the present study, ALPS indices were associated with sleep quality, whereas MFG‐centred connectivity was associated with addiction severity. This pattern suggests that sleep‐related glymphatic alterations and prefrontal network changes may contribute to IGD through partially distinct but potentially interacting pathways.

Based on these findings, we propose a cautious working model. In IGD, poorer sleep quality may be associated with reduced glymphatic system–related function, reflected by lower ALPS indices. This alteration may contribute to an unfavourable neural microenvironment. At the same time, altered MFG‐centred functional connectivity may reflect dysregulation of prefrontal control systems. These changes may be related to impaired cognitive control and greater addiction severity. Together, these findings support a potential sleep–glymphatic–prefrontal network framework for understanding the neurobiological mechanisms of IGD. This model remains hypothetical and requires confirmation in future longitudinal and interventional studies. Still, it provides a useful framework for understanding how sleep disturbance, glymphatic system–related alterations and prefrontal network dysfunction may jointly contribute to IGD.

Several implications can be drawn from this work. First, sleep quality should be considered an important clinical dimension in IGD rather than a secondary symptom alone. Second, DTI‐ALPS may be useful for exploring glymphatic system–related alterations in behavioural addiction. Third, MFG‐centred functional connectivity may help characterize prefrontal network changes associated with addiction severity. Future studies should include larger samples, objective sleep measurements and longitudinal follow‐up. Such work will be important for determining whether improving sleep quality can influence glymphatic system–related function and prefrontal connectivity in individuals with IGD.

## 5 Limitations

This study has several limitations. First, its cross‐sectional design precludes causal inference; longitudinal studies are needed to track dynamic ALPS‐FC relationships. Second, the DTI‐ALPS index is an indirect surrogate, requiring validation against direct measures like Aβ‐PET. Third, missing DTI data in some cases reduced the sample size for ALPS analysis, potentially limiting statistical power. Future work should ensure complete multimodal data acquisition and validate these findings in larger cohorts. Future research should (1) validate this ‘FC‐ALPS’ model in broader clinical populations, (2) explore the differential effects of specific sleep stages (e.g., REM vs. slow‐wave sleep) on ALPS indices and FC patterns and (3) develop and test targeted interventions to enhance glymphatic clearance.

## 6 Conclusion

By integrating DTI‐ALPS with MFG‐centred resting‐state functional connectivity analysis, this study provides multimodal neuroimaging evidence that IGD is associated with reduced glymphatic system–related function and altered prefrontal network connectivity. Lower ALPS indices were related to poorer sleep quality, whereas stronger MFG‐centred functional connectivity was associated with greater addiction severity. These findings support a potential sleep–glymphatic–prefrontal network framework for understanding the neurobiological mechanisms of IGD. Future longitudinal and interventional studies are needed to clarify the directionality of these associations and to determine whether improving sleep quality can influence glymphatic system–related function and prefrontal connectivity in individuals with IGD.

## Author Contributions

**Jiawen Tian:** conceptualization, methodology, formal analysis, visualization, writing – original draft. **Jiawen Tian:** methodology, formal analysis, visualization, writing – review and editing. **Xinyu Wang:** investigation, software, formal analysis, writing – review and editing. **Hongyu Zhang:** investigation, formal analysis, visualization, writing – review and editing. **Longyao Ma:** investigation, software, formal analysis, writing – review and editing. **Bohui Mei:** investigation, software, visualization, writing – review and editing. **Mengzhe Zhang:** data curation, software, validation, writing – review and editing. **Yan Lang:** data curation, software, validation, writing – review and editing. **Yarui Wei:** methodology, data curation, validation, writing – review and editing. **Shaoqiang Han:** conceptualization, supervision, project administration, validation, writing – review and editing. **Qingqing Lv:** methodology, data curation, validation, writing – review and editing. **Yong Zhang:** conceptualization, funding acquisition, project administration, resources, supervision, writing – review and editing. All authors contributed to the article and approved the submitted version.

## Funding

The authors declare that financial support was received for the research and/or publication of this article. This work was supported by the National Natural Science Foundation of China (Grant No. 82471962), the Scientific Research and Innovation Team of the First Affiliated Hospital of Zhengzhou University (Grant No. QNCXTD2023007) and the Henan Provincial Young and Middle‐aged Health Science and Technology Innovation Talent Support Program (Grant No. LJRC2025008).

## Ethics Statement

The studies involving human participants were reviewed and approved by the Ethics Committee of the First Affiliated Hospital of Zhengzhou University. All participants or their legal guardians provided written informed consent after being fully informed of the purpose and procedures of the study. The study was conducted in accordance with the Declaration of Helsinki.

## Conflicts of Interest

The authors declare no conflicts of interest.

## Data Availability

The datasets generated and/or analysed during the current study are not publicly available due to privacy and ethical restrictions but are available from the corresponding author on reasonable request.
